# Hexaaqua­zinc(II) bis­(2,4,5-tricarboxybenzoate) 4,5-diaza­fluoren-9-one disolvate dihydrate

**DOI:** 10.1107/S1600536810021641

**Published:** 2010-06-16

**Authors:** Gui-Fu Yang, Ya-Hui Zhao

**Affiliations:** a Computer School, Northeast Normal University, Changchun 130024, People’s Republic of China; b College Urban and Environmental Sciences, Northeast Normal University, Changchun 130024, People’s Republic of China

## Abstract

The asymmetric unit of the title complex, [Zn(H_2_O)_6_](C_10_H_5_O_8_)_2_·2C_11_H_6_N_2_O·2H_2_O, contains one half of the complex cation with the Zn^II^ ion located on an inversion center, a monovalent 2,4,5-tricarboxybenzoate (1,2,4,5-BTC) counter-anion, a 4,5-diaza­fluoren-9-one (DAFO) mol­ecule and an uncoordinated water mol­ecule. In the crystal structure, O—H⋯O and O—H⋯N hydrogen bonds link the cations, anions and water mol­ecules into a three-dimensional network.

## Related literature

For ZnII complexes, see: Rochon & Massarweh (2000 ▶); Si *et al.* (2003 ▶). For a related structure, see: Zhu *et al.* (2009 ▶).
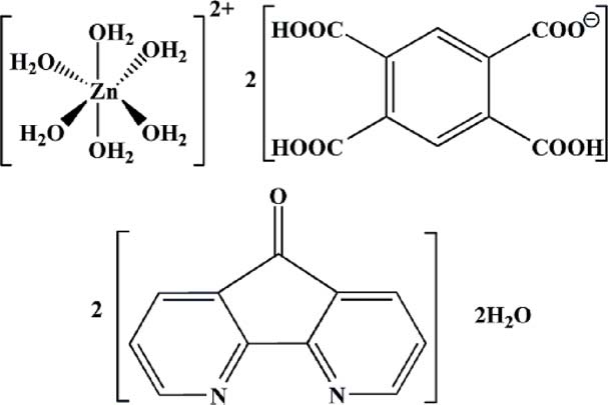


## Experimental

### 

#### Crystal data

[Zn(H_2_O)_6_](C_10_H_5_O_8_)_2_·2C_11_H_6_N_2_O·2H_2_O
*M* *_r_* = 1080.13
Triclinic, 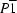

*a* = 8.380 (5) Å
*b* = 9.757 (5) Å
*c* = 14.107 (5) Å
α = 77.964 (5)°
β = 77.709 (5)°
γ = 89.948 (5)°
*V* = 1101.1 (9) Å^3^
*Z* = 1
Mo *K*α radiation
μ = 0.66 mm^−1^
*T* = 293 K
0.28 × 0.23 × 0.19 mm

#### Data collection

Rigaku R-AXIS RAPID diffractometer
Absorption correction: multi-scan (*ABSCOR*; Higashi, 1995 ▶) *T*_min_ = 0.836, *T*_max_ = 0.885
6855 measured reflections
5060 independent reflections
4670 reflections with *I* > 2σ(*I*)
*R*_int_ = 0.014

#### Refinement

*R*[*F* ^2^ > 2σ(*F* ^2^)] = 0.031
*wR*(*F* ^2^) = 0.086
*S* = 1.06
5060 reflections
343 parameters
H atoms treated by a mixture of independent and constrained refinement
Δρ_max_ = 0.28 e Å^−3^
Δρ_min_ = −0.34 e Å^−3^

### 

Data collection: *PROCESS-AUTO* (Rigaku, 1998 ▶); cell refinement: *PROCESS-AUTO*; data reduction: *PROCESS-AUTO*; program(s) used to solve structure: *SHELXS97* (Sheldrick, 2008 ▶); program(s) used to refine structure: *SHELXL97* (Sheldrick, 2008 ▶); molecular graphics: *SHELXTL-Plus* (Sheldrick, 2008 ▶); software used to prepare material for publication: *SHELXL97*.

## Supplementary Material

Crystal structure: contains datablocks I, global. DOI: 10.1107/S1600536810021641/er2077sup1.cif

Structure factors: contains datablocks I. DOI: 10.1107/S1600536810021641/er2077Isup2.hkl

Additional supplementary materials:  crystallographic information; 3D view; checkCIF report

## Figures and Tables

**Table:** 

Zn1—O9	2.0550 (15)
Zn1—O10	2.0712 (13)
Zn1—O11	2.0755 (12)

**Table:** 

O9^i^—Zn1—O10	89.81 (6)
O9—Zn1—O10	90.19 (6)
O9^i^—Zn1—O11	86.65 (5)
O9—Zn1—O11	93.35 (5)
O10^i^—Zn1—O11	89.23 (6)
O10—Zn1—O11	90.77 (6)

Symmetry code: (i) 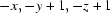.

**Table 2:** Hydrogen-bond geometry (Å, °)

*D*—H⋯*A*	*D*—H	H⋯*A*	*D*⋯*A*	*D*—H⋯*A*
O1*W*—H1*A*⋯O12^ii^	0.86	2.12	2.8342 (19)	141
O1*W*—H1*B*⋯O5^iii^	0.89	1.94	2.815 (2)	168
O9—H9*A*⋯O1*W*^ii^	0.75	2.13	2.8526 (19)	161
O9—H9*B*⋯O5^iii^	0.83	1.87	2.6929 (18)	177
O10—H10*A*⋯O1^iv^	0.82	1.96	2.7860 (18)	174
O11—H11*B*⋯N1^v^	0.80	2.10	2.880 (2)	166
O11—H11*A*⋯O6^iii^	0.76	2.05	2.7725 (17)	160
O10—H10*B*⋯N2^vi^	0.88	1.88	2.747 (2)	171
O3—H3*O*⋯O6^vii^	0.94 (3)	1.55 (3)	2.4883 (18)	173 (3)
O2—H2*O*⋯O1*W*	0.86 (3)	1.83 (3)	2.6747 (19)	167 (2)
O8—H8*O*⋯O4^viii^	0.85 (3)	1.83 (3)	2.670 (2)	171 (3)

Symmetry codes: (ii) 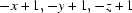; (iii) 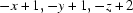; (iv) 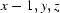; (v) 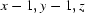; (vi) 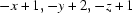; (vii) 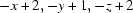; (viii) 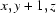.

**Fig. 1.**
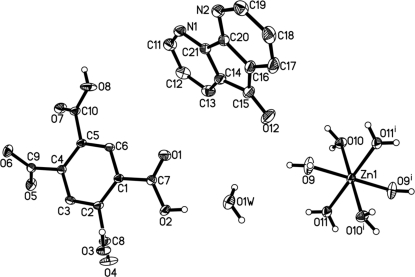
View of the local coordination of ZnII with the atomic numbering scheme. Displacement ellipsoids are drawn at the 30% probability level.

**Fig. 2.**
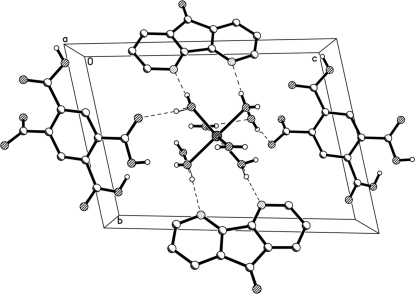
A packing diagram for the three-dimensional supramolecular framework via O—H···O interactions. The view direction is parallel to the a axis. Hydrogen bonds are indicated by dashed lines.

## supplementary crystallographic information

### Comment

Single-crystal X-ray diffraction analyses revealed Zn^II^ is hexa-coordinated and exhibits octahedral coordintion environment supplied by six water molecules (Fig. 1). The O atoms from four coordinated water molecules in the equatorial plane around the Zn^II^ ion form a slightly distorted square-planar arrangement with an average Zn—O bond length of 2.073 (1) Å; the slightly distorted octahedral coordination is completed by the other O atoms at a slightly shorter distance [2.055 (2) Å] in the axial positions. benzene-1,2,4,5-tetracarboxylate (1,2,4,5-BTC) counter-anion, and DAFO molecule are both uncoordinated. Intermolecular hydrogen bonds, O—H···O and O—H···N, extend the ion complex into a three-dimensional supramolecular network structure (Fig. 2, Table 1).

### Experimental

Zinc(II) acetate dihydrate (0.066 g, 0.3 mol), benzene-1,2,4,5-tetracarboxylate (0.055 g, 0.2 mmol), 4, 5-diazafluoren-9-one (0.036 g, 0.2 mmol), sodium hydroxide (0.016 g, 0.4 mmol) and water (14 ml) were placed in a 23 ml Teflon-lined autoclave, and the autoclave was heated at 423 K for 3 d. After cooling slowly to room temperature at a rate of 10 K h^-1^, colorless crystals were obtained.

### Refinement

C-bound H atoms were treated as riding, with C—H = 0.93Å and *U*_iso_(H) = 1.2 times *U*_eq_(C). O-bound H atoms were located in a difference Fourier map and refined as riding in their as-found relative positions; *U*_iso_(H) = 1.5*U*_eq_(O).

### Crystal data

**Table:**

[Zn(H_2_O)_6_](C_10_H_5_O_8_)_2_·2C_11_H_6_N_2_O·2H_2_O	*Z* = 1
*M**_r_* = 1080.13	*F*(000) = 556
Triclinic, *P*1	*D*_x_ = 1.629 Mg m^−^^3^
*a* = 8.380 (5) Å	Mo *K*α radiation, λ = 0.71069 Å
*b* = 9.757 (5) Å	Cell parameters from 6265 reflections
*c* = 14.107 (5) Å	θ = 2.1–28.2°
α = 77.964 (5)°	µ = 0.66 mm^−^^1^
β = 77.709 (5)°	*T* = 293 K
γ = 89.948 (5)°	Block, colorless
*V* = 1101.1 (9) Å^3^	0.28 × 0.23 × 0.19 mm

### Data collection

**Table:**

Rigaku R-AXIS RAPID diffractometer	5060 independent reflections
Radiation source: fine-focus sealed tube	4670 reflections with *I* > 2σ(*I*)
graphite	*R*_int_ = 0.014
Detector resolution: 10 pixels mm^-1^	θ_max_ = 28.3°, θ_min_ = 1.5°
ω scan	*h* = −11→4
Absorption correction: multi-scan (*ABSCOR*; Higashi, 1995)	*k* = −12→12
*T*_min_ = 0.836, *T*_max_ = 0.885	*l* = −18→16
6855 measured reflections	

### Refinement

**Table:**

Refinement on *F*^2^	Primary atom site location: structure-invariant direct methods
Least-squares matrix: full	Secondary atom site location: difference Fourier map
*R*[*F*^2^ > 2σ(*F*^2^)] = 0.031	Hydrogen site location: inferred from neighbouring sites
*wR*(*F*^2^) = 0.086	H atoms treated by a mixture of independent and constrained refinement
*S* = 1.06	*w* = 1/[σ^2^(*F*_o_^2^) + (0.0461*P*)^2^ + 0.2811*P*] where *P* = (*F*_o_^2^ + 2*F*_c_^2^)/3
5060 reflections	(Δ/σ)_max_ = 0.001
343 parameters	Δρ_max_ = 0.28 e Å^−^^3^
0 restraints	Δρ_min_ = −0.34 e Å^−^^3^

### Special details

**Table:**

Geometry. All e.s.d.'s (except the e.s.d. in the dihedral angle between two l.s. planes) are estimated using the full covariance matrix. The cell e.s.d.'s are taken into account individually in the estimation of e.s.d.'s in distances, angles and torsion angles; correlations between e.s.d.'s in cell parameters are only used when they are defined by crystal symmetry. An approximate (isotropic) treatment of cell e.s.d.'s is used for estimating e.s.d.'s involving l.s. planes.
Refinement. Refinement of *F*^2^ against ALL reflections. The weighted *R*-factor *wR* and goodness of fit *S* are based on *F*^2^, conventional *R*-factors *R* are based on *F*, with *F* set to zero for negative *F*^2^. The threshold expression of *F*^2^ > σ(*F*^2^) is used only for calculating *R*-factors(gt) *etc*. and is not relevant to the choice of reflections for refinement. *R*-factors based on *F*^2^ are statistically about twice as large as those based on *F*, and *R*- factors based on ALL data will be even larger.

### Fractional atomic coordinates and isotropic or equivalent isotropic displacement parameters (Å^2^)

**Table:**

	*x*	*y*	*z*	*U*_iso_*/*U*_eq_	
Zn1	0.0000	0.5000	0.5000	0.02688 (8)	
O1	0.70290 (17)	0.59156 (12)	0.76405 (8)	0.0414 (3)	
O2	0.64715 (15)	0.36226 (11)	0.82062 (9)	0.0356 (2)	
O3	0.89652 (15)	0.20199 (11)	0.90798 (9)	0.0374 (3)	
O4	0.68585 (18)	0.13427 (11)	1.03103 (9)	0.0510 (4)	
O5	0.72889 (14)	0.45006 (13)	1.28469 (8)	0.0394 (3)	
O6	0.93948 (14)	0.60062 (13)	1.21458 (8)	0.0371 (3)	
O7	0.68773 (16)	0.79402 (12)	1.15402 (8)	0.0408 (3)	
O8	0.7470 (2)	0.88143 (12)	0.99083 (9)	0.0521 (4)	
O12	0.55282 (18)	0.75220 (15)	0.47805 (12)	0.0579 (4)	
N1	0.76786 (17)	1.09551 (14)	0.61753 (10)	0.0354 (3)	
N2	0.93283 (18)	1.14163 (15)	0.39355 (10)	0.0372 (3)	
C1	0.72961 (16)	0.49108 (14)	0.92716 (10)	0.0235 (3)	
C2	0.76421 (17)	0.37449 (13)	0.99478 (10)	0.0241 (3)	
C3	0.78982 (18)	0.39158 (14)	1.08567 (10)	0.0269 (3)	
H3	0.8115	0.3136	1.1309	0.032*	
C4	0.78401 (16)	0.52197 (14)	1.11107 (10)	0.0239 (3)	
C5	0.74847 (17)	0.63815 (13)	1.04361 (10)	0.0240 (3)	
C6	0.72178 (17)	0.62128 (14)	0.95285 (10)	0.0254 (3)	
H6	0.6981	0.6990	0.9082	0.030*	
C7	0.69319 (17)	0.48682 (14)	0.82862 (10)	0.0267 (3)	
C8	0.77793 (19)	0.22755 (14)	0.97678 (10)	0.0293 (3)	
C9	0.81864 (17)	0.52666 (14)	1.21153 (10)	0.0260 (3)	
C10	0.72500 (18)	0.77881 (14)	1.07017 (11)	0.0280 (3)	
C11	0.6757 (2)	1.05207 (18)	0.71039 (13)	0.0422 (4)	
H11	0.6890	1.1019	0.7579	0.051*	
C12	0.5640 (2)	0.9399 (2)	0.74011 (14)	0.0462 (4)	
H12	0.5047	0.9166	0.8054	0.055*	
C13	0.5404 (2)	0.86182 (18)	0.67221 (14)	0.0432 (4)	
H13	0.4665	0.7850	0.6900	0.052*	
C14	0.63219 (19)	0.90423 (16)	0.57714 (13)	0.0349 (3)	
C15	0.6374 (2)	0.84773 (18)	0.48618 (14)	0.0401 (4)	
C16	0.7622 (2)	0.93733 (17)	0.40690 (13)	0.0371 (3)	
C17	0.8169 (2)	0.9358 (2)	0.30764 (14)	0.0477 (4)	
H17	0.7787	0.8677	0.2796	0.057*	
C18	0.9305 (3)	1.0392 (2)	0.25193 (14)	0.0503 (5)	
H18	0.9710	1.0421	0.1848	0.060*	
C19	0.9840 (2)	1.1388 (2)	0.29631 (13)	0.0456 (4)	
H19	1.0600	1.2081	0.2569	0.055*	
C20	0.82315 (18)	1.04143 (15)	0.44622 (12)	0.0315 (3)	
C21	0.74262 (18)	1.01997 (15)	0.55338 (11)	0.0304 (3)	
O1W	0.54813 (14)	0.40373 (14)	0.64908 (9)	0.0427 (3)	
H1A	0.5031	0.3305	0.6388	0.064*	
H1B	0.4692	0.4612	0.6658	0.064*	
O9	0.21520 (14)	0.55344 (16)	0.53380 (9)	0.0485 (3)	
H9A	0.2906	0.5561	0.4935	0.073*	
H9B	0.2308	0.5549	0.5895	0.073*	
O10	−0.11749 (14)	0.65168 (12)	0.56875 (8)	0.0378 (3)	
H10A	−0.1723	0.6286	0.6259	0.057*	
H11B	−0.0987	0.2735	0.6240	0.057*	
O11	−0.06978 (15)	0.34898 (12)	0.63034 (8)	0.0389 (3)	
H11A	−0.0226	0.3459	0.6709	0.058*	
H10B	−0.0502	0.7156	0.5757	0.058*	
H3O	0.957 (3)	0.281 (3)	0.865 (2)	0.083 (9)*	
H2O	0.629 (3)	0.369 (3)	0.7620 (19)	0.063 (7)*	
H8O	0.732 (3)	0.959 (3)	1.0088 (19)	0.071 (8)*	

### Atomic displacement parameters (Å^2^)

**Table:**

	*U*^11^	*U*^22^	*U*^33^	*U*^12^	*U*^13^	*U*^23^
Zn1	0.03260 (13)	0.02768 (13)	0.02240 (12)	0.00010 (9)	−0.00775 (9)	−0.00810 (9)
O1	0.0733 (8)	0.0291 (6)	0.0246 (5)	0.0058 (5)	−0.0168 (5)	−0.0054 (4)
O2	0.0530 (7)	0.0292 (5)	0.0304 (6)	−0.0027 (5)	−0.0169 (5)	−0.0114 (4)
O3	0.0468 (6)	0.0257 (5)	0.0383 (6)	0.0047 (5)	0.0001 (5)	−0.0130 (5)
O4	0.0778 (9)	0.0201 (5)	0.0433 (7)	−0.0077 (5)	0.0147 (6)	−0.0091 (5)
O5	0.0452 (6)	0.0485 (7)	0.0221 (5)	−0.0104 (5)	−0.0067 (4)	−0.0030 (5)
O6	0.0418 (6)	0.0449 (6)	0.0264 (5)	−0.0096 (5)	−0.0109 (4)	−0.0080 (5)
O7	0.0630 (8)	0.0324 (6)	0.0323 (6)	0.0055 (5)	−0.0105 (5)	−0.0182 (5)
O8	0.1023 (11)	0.0172 (5)	0.0347 (6)	0.0055 (6)	−0.0075 (7)	−0.0088 (5)
O12	0.0530 (8)	0.0509 (8)	0.0825 (11)	−0.0060 (6)	−0.0266 (7)	−0.0304 (7)
N1	0.0454 (7)	0.0281 (6)	0.0353 (7)	0.0025 (5)	−0.0097 (6)	−0.0120 (5)
N2	0.0451 (7)	0.0342 (7)	0.0343 (7)	0.0042 (6)	−0.0091 (6)	−0.0114 (5)
C1	0.0307 (6)	0.0199 (6)	0.0202 (6)	0.0004 (5)	−0.0046 (5)	−0.0060 (5)
C2	0.0326 (7)	0.0175 (6)	0.0224 (6)	0.0002 (5)	−0.0041 (5)	−0.0065 (5)
C3	0.0387 (7)	0.0195 (6)	0.0227 (6)	0.0041 (5)	−0.0080 (5)	−0.0034 (5)
C4	0.0293 (6)	0.0231 (6)	0.0200 (6)	0.0007 (5)	−0.0053 (5)	−0.0066 (5)
C5	0.0322 (7)	0.0189 (6)	0.0221 (6)	0.0007 (5)	−0.0053 (5)	−0.0077 (5)
C6	0.0370 (7)	0.0187 (6)	0.0210 (6)	0.0027 (5)	−0.0071 (5)	−0.0045 (5)
C7	0.0333 (7)	0.0259 (7)	0.0227 (7)	0.0036 (5)	−0.0059 (5)	−0.0098 (5)
C8	0.0452 (8)	0.0177 (6)	0.0257 (7)	0.0021 (5)	−0.0066 (6)	−0.0072 (5)
C9	0.0330 (7)	0.0258 (6)	0.0219 (6)	0.0045 (5)	−0.0081 (5)	−0.0089 (5)
C10	0.0378 (7)	0.0200 (6)	0.0281 (7)	0.0012 (5)	−0.0077 (6)	−0.0091 (5)
C11	0.0540 (10)	0.0377 (9)	0.0363 (9)	0.0072 (7)	−0.0071 (7)	−0.0140 (7)
C12	0.0472 (10)	0.0423 (9)	0.0427 (10)	0.0068 (7)	0.0013 (7)	−0.0061 (7)
C13	0.0366 (8)	0.0335 (8)	0.0564 (11)	0.0027 (6)	−0.0066 (7)	−0.0062 (7)
C14	0.0323 (7)	0.0285 (7)	0.0480 (9)	0.0064 (6)	−0.0134 (6)	−0.0127 (6)
C15	0.0373 (8)	0.0347 (8)	0.0584 (11)	0.0077 (6)	−0.0223 (7)	−0.0204 (7)
C16	0.0394 (8)	0.0374 (8)	0.0447 (9)	0.0100 (6)	−0.0205 (7)	−0.0199 (7)
C17	0.0554 (11)	0.0535 (11)	0.0506 (11)	0.0162 (9)	−0.0277 (9)	−0.0311 (9)
C18	0.0615 (12)	0.0607 (12)	0.0353 (9)	0.0172 (9)	−0.0156 (8)	−0.0203 (8)
C19	0.0546 (10)	0.0469 (10)	0.0351 (9)	0.0076 (8)	−0.0080 (7)	−0.0099 (7)
C20	0.0364 (7)	0.0280 (7)	0.0360 (8)	0.0087 (6)	−0.0145 (6)	−0.0131 (6)
C21	0.0337 (7)	0.0255 (7)	0.0355 (8)	0.0073 (5)	−0.0111 (6)	−0.0108 (6)
O1W	0.0379 (6)	0.0548 (7)	0.0407 (7)	0.0006 (5)	−0.0088 (5)	−0.0221 (6)
O9	0.0346 (6)	0.0857 (10)	0.0278 (6)	−0.0111 (6)	−0.0068 (5)	−0.0177 (6)
O10	0.0448 (6)	0.0351 (6)	0.0336 (6)	0.0016 (5)	−0.0024 (5)	−0.0140 (5)
O11	0.0550 (7)	0.0349 (6)	0.0283 (6)	−0.0069 (5)	−0.0156 (5)	−0.0031 (4)

### Geometric parameters (Å, °)

**Table:**

Zn1—O9^i^	2.0550 (15)	C4—C9	1.5156 (19)
Zn1—O9	2.0550 (15)	C5—C6	1.3879 (19)
Zn1—O10^i^	2.0712 (13)	C5—C10	1.4983 (19)
Zn1—O10	2.0712 (13)	C6—H6	0.9300
Zn1—O11^i^	2.0755 (12)	C11—C12	1.376 (3)
Zn1—O11	2.0755 (12)	C11—H11	0.9300
O1—C7	1.2088 (18)	C12—C13	1.386 (3)
O2—C7	1.3097 (18)	C12—H12	0.9300
O2—H2O	0.86 (3)	C13—C14	1.375 (3)
O3—C8	1.2968 (18)	C13—H13	0.9300
O3—H3O	0.94 (3)	C14—C21	1.399 (2)
O4—C8	1.2119 (19)	C14—C15	1.491 (2)
O5—C9	1.2402 (18)	C15—C16	1.485 (3)
O6—C9	1.2573 (19)	C16—C17	1.381 (3)
O7—C10	1.1978 (18)	C16—C20	1.399 (2)
O8—C10	1.3169 (19)	C17—C18	1.376 (3)
O8—H8O	0.85 (3)	C17—H17	0.9300
O12—C15	1.210 (2)	C18—C19	1.383 (3)
N1—C21	1.329 (2)	C18—H18	0.9300
N1—C11	1.353 (2)	C19—H19	0.9300
N2—C20	1.327 (2)	C20—C21	1.489 (2)
N2—C19	1.354 (2)	O1W—H1A	0.8602
C1—C6	1.3897 (19)	O1W—H1B	0.8916
C1—C2	1.3974 (19)	O9—H9A	0.7509
C1—C7	1.4939 (19)	O9—H9B	0.8266
C2—C3	1.3871 (19)	O10—H10A	0.8240
C2—C8	1.5075 (19)	O10—H10B	0.8756
C3—C4	1.390 (2)	O11—H11B	0.8033
C3—H3	0.9300	O11—H11A	0.7581
C4—C5	1.3962 (19)		
			
O9^i^—Zn1—O9	180.00 (6)	O7—C10—O8	124.78 (13)
O9^i^—Zn1—O10^i^	90.19 (6)	O7—C10—C5	123.23 (13)
O9—Zn1—O10^i^	89.81 (6)	O8—C10—C5	111.97 (12)
O9^i^—Zn1—O10	89.81 (6)	N1—C11—C12	125.12 (17)
O9—Zn1—O10	90.19 (6)	N1—C11—H11	117.4
O10^i^—Zn1—O10	180.000 (1)	C12—C11—H11	117.4
O9^i^—Zn1—O11^i^	93.35 (5)	C11—C12—C13	119.65 (17)
O9—Zn1—O11^i^	86.65 (5)	C11—C12—H12	120.2
O10^i^—Zn1—O11^i^	90.77 (6)	C13—C12—H12	120.2
O10—Zn1—O11^i^	89.23 (6)	C14—C13—C12	116.35 (16)
O9^i^—Zn1—O11	86.65 (5)	C14—C13—H13	121.8
O9—Zn1—O11	93.35 (5)	C12—C13—H13	121.8
O10^i^—Zn1—O11	89.23 (6)	C13—C14—C21	120.15 (15)
O10—Zn1—O11	90.77 (6)	C13—C14—C15	131.05 (16)
O11^i^—Zn1—O11	180.0	C21—C14—C15	108.79 (15)
C7—O2—H2O	108.3 (17)	O12—C15—C16	127.32 (18)
C8—O3—H3O	116.1 (17)	O12—C15—C14	126.97 (18)
C10—O8—H8O	109.1 (18)	C16—C15—C14	105.65 (13)
C21—N1—C11	114.33 (14)	C17—C16—C20	119.59 (17)
C20—N2—C19	115.56 (15)	C17—C16—C15	131.56 (16)
C6—C1—C2	119.02 (12)	C20—C16—C15	108.80 (15)
C6—C1—C7	116.07 (12)	C18—C17—C16	117.23 (17)
C2—C1—C7	124.86 (12)	C18—C17—H17	121.4
C3—C2—C1	119.25 (12)	C16—C17—H17	121.4
C3—C2—C8	115.88 (12)	C17—C18—C19	119.64 (17)
C1—C2—C8	124.87 (12)	C17—C18—H18	120.2
C2—C3—C4	121.84 (12)	C19—C18—H18	120.2
C2—C3—H3	119.1	N2—C19—C18	124.07 (18)
C4—C3—H3	119.1	N2—C19—H19	118.0
C3—C4—C5	118.79 (12)	C18—C19—H19	118.0
C3—C4—C9	116.48 (12)	N2—C20—C16	123.90 (15)
C5—C4—C9	124.73 (12)	N2—C20—C21	127.60 (14)
C6—C5—C4	119.52 (12)	C16—C20—C21	108.50 (14)
C6—C5—C10	118.91 (12)	N1—C21—C14	124.40 (15)
C4—C5—C10	121.39 (12)	N1—C21—C20	127.36 (14)
C5—C6—C1	121.57 (12)	C14—C21—C20	108.24 (13)
C5—C6—H6	119.2	H1A—O1W—H1B	107.9
C1—C6—H6	119.2	Zn1—O9—H9A	115.7
O1—C7—O2	124.26 (14)	Zn1—O9—H9B	126.9
O1—C7—C1	121.57 (13)	H9A—O9—H9B	115.9
O2—C7—C1	114.14 (12)	Zn1—O10—H10A	119.5
O4—C8—O3	121.35 (13)	Zn1—O10—H10B	113.2
O4—C8—C2	119.60 (13)	H10A—O10—H10B	100.6
O3—C8—C2	118.84 (12)	Zn1—O11—H11B	115.8
O5—C9—O6	124.96 (13)	Zn1—O11—H11A	119.0
O5—C9—C4	116.40 (13)	H11B—O11—H11A	114.1
O6—C9—C4	118.52 (12)		
			
C6—C1—C2—C3	0.0 (2)	N1—C11—C12—C13	0.3 (3)
C7—C1—C2—C3	−177.52 (13)	C11—C12—C13—C14	−0.5 (3)
C6—C1—C2—C8	−179.46 (13)	C12—C13—C14—C21	0.3 (2)
C7—C1—C2—C8	3.0 (2)	C12—C13—C14—C15	−179.10 (16)
C1—C2—C3—C4	−0.8 (2)	C13—C14—C15—O12	3.2 (3)
C8—C2—C3—C4	178.71 (13)	C21—C14—C15—O12	−176.28 (17)
C2—C3—C4—C5	1.2 (2)	C13—C14—C15—C16	−179.47 (17)
C2—C3—C4—C9	−178.42 (13)	C21—C14—C15—C16	1.10 (17)
C3—C4—C5—C6	−0.7 (2)	O12—C15—C16—C17	−1.6 (3)
C9—C4—C5—C6	178.82 (13)	C14—C15—C16—C17	−178.92 (17)
C3—C4—C5—C10	174.23 (13)	O12—C15—C16—C20	175.75 (17)
C9—C4—C5—C10	−6.2 (2)	C14—C15—C16—C20	−1.62 (17)
C4—C5—C6—C1	0.0 (2)	C20—C16—C17—C18	−0.2 (2)
C10—C5—C6—C1	−175.12 (13)	C15—C16—C17—C18	176.91 (18)
C2—C1—C6—C5	0.4 (2)	C16—C17—C18—C19	−0.2 (3)
C7—C1—C6—C5	178.14 (12)	C20—N2—C19—C18	−0.7 (3)
C6—C1—C7—O1	20.5 (2)	C17—C18—C19—N2	0.6 (3)
C2—C1—C7—O1	−161.92 (15)	C19—N2—C20—C16	0.4 (2)
C6—C1—C7—O2	−157.94 (13)	C19—N2—C20—C21	−178.61 (15)
C2—C1—C7—O2	19.6 (2)	C17—C16—C20—N2	0.0 (2)
C3—C2—C8—O4	61.2 (2)	C15—C16—C20—N2	−177.65 (14)
C1—C2—C8—O4	−119.33 (18)	C17—C16—C20—C21	179.20 (14)
C3—C2—C8—O3	−113.59 (16)	C15—C16—C20—C21	1.52 (17)
C1—C2—C8—O3	65.9 (2)	C11—N1—C21—C14	−0.4 (2)
C3—C4—C9—O5	−57.77 (18)	C11—N1—C21—C20	179.48 (15)
C5—C4—C9—O5	122.67 (16)	C13—C14—C21—N1	0.2 (2)
C3—C4—C9—O6	118.48 (15)	C15—C14—C21—N1	179.69 (14)
C5—C4—C9—O6	−61.1 (2)	C13—C14—C21—C20	−179.72 (14)
C6—C5—C10—O7	152.54 (15)	C15—C14—C21—C20	−0.21 (17)
C4—C5—C10—O7	−22.5 (2)	N2—C20—C21—N1	−1.6 (3)
C6—C5—C10—O8	−26.2 (2)	C16—C20—C21—N1	179.28 (15)
C4—C5—C10—O8	158.82 (15)	N2—C20—C21—C14	178.31 (15)
C21—N1—C11—C12	0.2 (3)	C16—C20—C21—C14	−0.83 (17)

Symmetry codes: (i) −*x*, −*y*+1, −*z*+1.

### Hydrogen-bond geometry (Å, °)

**Table:**

*D*—H···*A*	*D*—H	H···*A*	*D*···*A*	*D*—H···*A*
O1W—H1A···O12^ii^	0.86	2.12	2.8342 (19)	141
O1W—H1B···O5^iii^	0.89	1.94	2.815 (2)	168
O9—H9A···O1W^ii^	0.75	2.13	2.8526 (19)	161
O9—H9B···O5^iii^	0.83	1.87	2.6929 (18)	177
O10—H10A···O1^iv^	0.82	1.96	2.7860 (18)	174
O11—H11B···N1^v^	0.80	2.10	2.880 (2)	166
O11—H11A···O6^iii^	0.76	2.05	2.7725 (17)	160
O10—H10B···N2^vi^	0.88	1.88	2.747 (2)	171
O3—H3O···O6^vii^	0.94 (3)	1.55 (3)	2.4883 (18)	173 (3)
O2—H2O···O1W	0.86 (3)	1.83 (3)	2.6747 (19)	167 (2)
O8—H8O···O4^viii^	0.85 (3)	1.83 (3)	2.670 (2)	171 (3)

Symmetry codes: (ii) −*x*+1, −*y*+1, −*z*+1; (iii) −*x*+1, −*y*+1, −*z*+2; (iv) *x*−1, *y*, *z*; (v) *x*−1, *y*−1, *z*; (vi) −*x*+1, −*y*+2, −*z*+1; (vii) −*x*+2, −*y*+1, −*z*+2; (viii) *x*, *y*+1, *z*.
